# Microbial Matryoshka: Addressing the Relationship between Pathogenic Flagellated Protozoans and Their RNA Viral Endosymbionts (Family *Totiviridae*)

**DOI:** 10.3390/vetsci11070321

**Published:** 2024-07-17

**Authors:** Alexandra Ibañez-Escribano, Maria Teresa Gomez-Muñoz, Marta Mateo, Cristina Fonseca-Berzal, Esperanza Gomez-Lucia, Raquel Garcia Perez, Jose M. Alunda, Javier Carrion

**Affiliations:** 1 Department of Microbiology and Parasitology, Faculty of Pharmacy, Complutense University of Madrid, 28040 Madrid, Spain; alexandraibanez@ucm.es (A.I.-E.); mmateo14@ucm.es (M.M.); crfonseca@ucm.es (C.F.-B.); 2 ICPVet Research Group, Department of Animal Health, Faculty of Veterinary Medicine, Complutense University of Madrid, 28040 Madrid, Spain; mariateg@ucm.es (M.T.G.-M.); raquel16@ucm.es (R.G.P.); jmalunda@ucm.es (J.M.A.); 3 Research Institute Hospital 12 de Octubre, 28041 Madrid, Spain; 4 Animal Viruses Research Group, Department of Animal Health, Faculty of Veterinary Medicine, Complutense University of Madrid, 28040 Madrid, Spain; duato@ucm.es

**Keywords:** Totiviridae, Giardiavirus, Trichomonasvirus, Leishmaniavirus, RNA virus, viral endosymbiont

## Abstract

**Simple Summary:**

Giardiosis, trichomonosis, leishmaniosis, and trypanosomosis are parasitic diseases caused by flagellated protozoa that have a major global health impact, and their control is a priority action line in the agenda of the current One Health Program. The pathogens causing these diseases can establish an endosymbiotic relationship with RNA viruses of the *Totiviridae* family that can alter the course of the final infection in a mammal. To easily understand the sequence of interactions that occur between the agents involved, from a structural point of view, we can imagine a “matryoshka”-type infection model, wherein the virus represents the smallest matryoshka infecting the flagellated protozoan, which represents the medium matryoshka infecting the mammal, the largest matryoshka. In this manuscript, we will review the available information on the complications generated, such as the aggravation of pathogenesis or treatment failures, because of the established association between these flagellated pathogens and their respective endosymbiont viruses. Accurate diagnosis is required to detect these situations of endosymbiont co-infection and to be able to apply tailor-made treatments that target both the flagellated parasite and the virus that hides inside it. Taken together, these approaches will allow us to achieve and optimize appropriate sanitary control strategies.

**Abstract:**

Three genera of viruses of the family *Totiviridae* establish endosymbiotic associations with flagellated protozoa responsible for parasitic diseases of great impact in the context of One Health. *Giardiavirus*, *Trichomonasvirus*, and *Leishmaniavirus* infect the protozoa *Giardia* sp., *Trichomonas vaginalis*, and *Leishmania* sp., respectively. In the present work, we review the characteristics of the endosymbiotic relationships established, the advantages, and the consequences caused in mammalian hosts. Among the common characteristics of these double-stranded RNA viruses are that they do not integrate into the host genome, do not follow a lytic cycle, and do not cause cytopathic effects. However, in cases of endosymbiosis between *Leishmaniavirus* and *Leishmania* species from the Americas, and between *Trichomonasvirus* and *Trichomonas vaginalis*, it seems that it can alter their virulence (degree of pathogenicity). In a mammalian host, due to TLR3 activation of immune cells upon the recognition of viral RNA, uncontrolled inflammatory signaling responses are triggered, increasing pathological damage and the risk of failure of conventional standard treatment. Endosymbiosis with *Giardiavirus* can cause the loss of intestinal adherence of the protozoan, resulting in a benign disease. The current knowledge about viruses infecting flagellated protozoans is still fragmentary, and more research is required to unravel the intricacies of this three-way relationship. We need to develop early and effective diagnostic methods for further development in the field of translational medicine. Taking advantage of promising biotechnological advances, the aim is to develop ad hoc therapeutic strategies that focus not only on the disease-causing protozoan but also on the virus.

## 1. Introduction

Parasitic diseases caused by protozoa are of enormous relevance to human and animal health in the One Health context. Most of these diseases are distributed worldwide, many with special incidence in areas related to poverty and a lack of adequate hygienic conditions. It is in these cases where assistance through vaccines and treatments remains neglected by health authorities and pharmaceutical companies. In addition, these protozoa have developed over the course of evolution successful strategies to escape from the immune system, which further complicates the achievement of vaccines and therapies [1].

This review will focus mainly on medically important diseases caused by protozoan parasites (giardiosis, trichomonosis, leishmaniosis, and trypanosomosis), in which cases of co-infection with endosymbiont viruses belonging to the family *Totiviridae* have been described. Parasitic protozoan viruses (PPVs) of the *Totiviridae* family share common characteristics: an icosahedral shape, a non-enveloped form, an average size of about 40 nanometers, and a viral genome made up of double-stranded RNA (dsRNA), with a size of 4–7 kb that encodes the capsid protein (CP), as well as the RNA-dependent RNA polymerase (RdRP), which is its own RNA polymerase that catalyzes the multiplication of viral RNA. Furthermore, the integration of the viral genome into the protozoan genome has not been described [2]. The transmission of the virus occurs vertically during the bipartition of the protozoan, although horizontal transmission has also been described [3]. Another common characteristic is that the virus does not exert cytopathic effects on the flagellated protozoan cell, within which it multiplies in a controlled manner following a non-lytic viral cycle. The *Totiviridae* family is composed of five genera: the genera *Victorivirus* and *Totivirus*, which infect fungi, and the genera *Giardiavirus* (GLV: *Giardia lamblia virus*), *Trichomonasvirus* (TVV: *Trichomonas vaginalis virus*), and *Leishmaniavirus* (LRV: *Leishmania RNA virus*), which infect the corresponding protozoan genera [4]. To easily understand the sequence of interactions that occur between the agents involved, from a structural point of view, a “matryoshka” model of infection can be contemplated, a term that other authors have previously coined to refer to RNA viruses infecting other protozoa of the genus *Plasmodium* [5], among others. Thus, using this conceptual model of Russian dolls, the virus represents the smallest matryoshka infecting the protozoan, which represents the medium-sized matryoshka infecting the mammalian host, the largest matryoshka, included in ecosystems with interactions between individuals and with other animal species. Parasitic protozoan viruses differ from common mammalian viruses in that they do not cause host cell lysis [6], and, in addition, they usually have a smooth surface that lacks the conventional viral receptor-clustering structure, making it difficult to recognize the invasion mechanisms used by protozoan viruses (except for GLV, in which a receptor has been described) [2]. Recent research has suggested that the hydrophobic region of the protozoan viruses’ surface mediates the viral invasion of host cells. The protozoan proteins and organelles used by PPVs to complete their life cycles, including self-replication and assembly mechanisms, are also unknown [7]. One of the objectives of this review is to clarify whether the presence of viruses in these protozoa has a modulating effect on their virulence. Describing in detail the different possible scenarios proposed by several studies is also an objective of this review. Some of these viruses seem to have no effect on their protozoan hosts, but others increase the virulent phenotype of the protozoan infection and contribute to intensifying the disease in humans. Thus, several studies have reported cases in which the corresponding *Totiviridae* viral symbionts can promote a hypervirulent phenotype of parasitic infection caused by the genera *Trichomonas* and *Leishmania*, while in the genus *Giardia,* they could have a neutral effect or cause a hypovirulent phenotype [8]. Further consequences of this endosymbiont relationship occur when an incomplete diagnosis has been made in a sick patient, in which the virus remains unnoticed and the conventional treatment (i.e., metronidazole against giardiosis and trichomonosis, or antimonial compounds against cutaneous leishmaniosis) destroy the protozoan cell. This allows the release of the viral genetic material that interacts with the TLR3 of the mammalian cells and triggers the hyperinflammatory signaling cascade [9,10,11]. TLRs are intracellular or surface-related host sentinels and links between innate and adaptive immunity, recognizing pathogen-associated molecular patterns (PAMPs) via PRRs (pattern recognition receptors). The interaction of a PPV in the final mammalian host may indirectly favor the ability of the protozoan to evade the immune response. Deciphering these mechanisms is crucial to avoid therapeutic failures with conventional antiprotozoal compounds [7]. In the case of *Giardiavirus*, due to its high thermoresistance, the viral material is not usually released, minimizing its possible interaction with the TLR3 of mammalian cells [7]. However, in the case of *Trichomonasvirus*, other consequences can occur [12]. In *Totiviridae*, viral infection does not alter the growth of their hosts as described in the ICTV web resource (International Committee on Taxonomy of Viruses: https://ictv.global/report_9th/dsRNA/Totiviridae accessed on 1 May 2024). Nevertheless, it is detailed below how GLV can modulate the growth of the protozoa.

## 2. *Totiviridae* Family: Viral and Genomic Structure, Cycle, and Other Details

Virions are non-enveloped, with an icosahedral capsid of 30–40 nm in diameter composed of dimers of a single type of protein. This capsid contains pores, clefts, or channels in the five-fold vertices [13,14], presumably for the release of viral mRNA [15,16]. GLV virions are substantially more thermo-tolerant than other species [2]. Capsids enclose a double-stranded RNA (dsRNA), which would be equivalent to that of the family *Reoviridae* in vertebrates and invertebrates, but unlike Reoviruses, it is a non-segmented molecule of 4.6 to 6.7 kb long.

**Figure 1:**
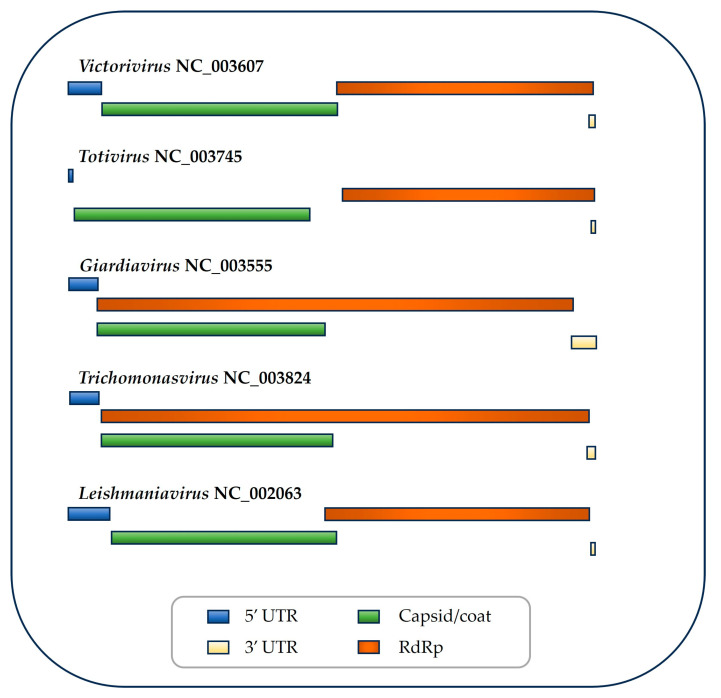
**Genomic organization of the five genera of *Totiviridae*.** GenBank accession number of the type species is shown in each genus. ORF1 is shown in green. ORF2 is shown in red. UTR, untranslated region. RdRp, RNA-dependent RNA polymerase. This review focuses on the three genera that infect flagellated protozoans.

Each viral particle also contains 1–2 copies of a viral polymerase [15,17]. RNA has a dual function, as mRNA and as a replicative intermediary [18]. Single-stranded RNA (ssRNA) spans the entire length of the positive genomic strand, lacks a 5′ cap, and is not polyadenylated. The positive strand has two large open reading frames (ORFs), preceded by an internal ribosomal entry site (IRES) in the untranslated region (UTR) at the 5′ end (Figure 1) where translation is initiated [2]. ORF1 encodes the capsid protein (GAG, gp2, and CP) with a predicted size of 76–81 × 10^3^. ORF2 encodes the RNA-dependent RNA polymerase (RdRp, POL, and gp1). RdRp is the only universal “hallmark” gene of RNA viruses [19].

**Figure 2:**
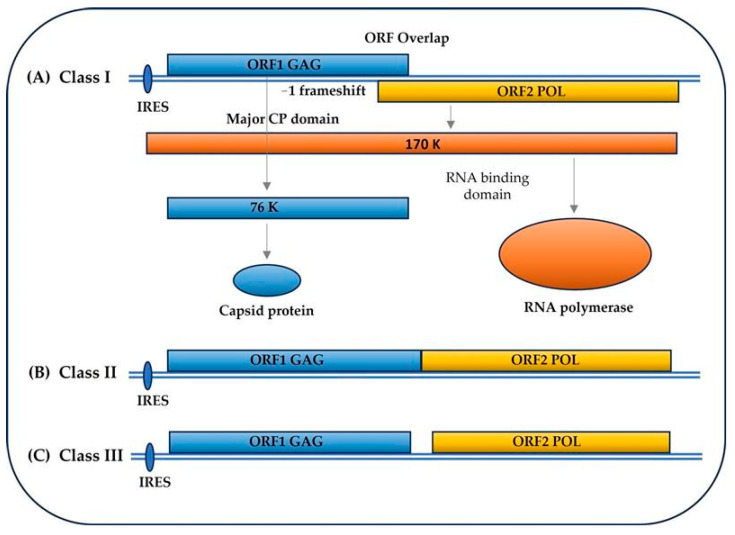
**Classes of *Totiviridae* according to the origin of the RdRp**. (**A**) Class I, fusion protein encoded in two different frames. (**B**) Class II, fusion protein encoded by the two adjacent ORFs in the same frame. (**C**) Class III, non-fusion protein encoded by ORF2. IRES, internal ribosome entry site, allowing initiation of translation by host translation machinery.

According to the generation of RdRp, there are three types of members in the family *Totiviridae* (Figure 2): in class I (e.g., *Leishmaniavirus* of the New World, *Giardiavirus,* or *Trichomonasvirus*), both ORFs overlap by 10–220 nt (16 to 130 nt in LRV1; 220 in GLV [20]); in class II, RdRp is formed by the fusion of ORF1 and ORF2 in the same frame; while in class III (e.g., LRV of the Old World, *Totivirus,* or *Victorivirus*), *pol* is a non-fusion protein in a separate ORF. In class I totiviruses, ORF1 and ORF2 experience a translational frameshift and encode together via −1 or +1 (−2 in *Trichomonasvirus* [15]) the putative RdRp as a fusion protein (*gag-pol* [21]), with a predicted Mr of 170–180 × 10^3^ [22]. This happens because the overlap region contains a slippery heptamer motif, an essential RNA pseudoknot shortly downstream of the site, a short spacer region with several stem-loops, and a coupled termination reinitiation mechanism [21,23,24]. These structures are not present in other *Totiviridae*, such as the New World *Leishmaniavirus*; consequently, there is no ribosomal frameshift. 

**Figure 3:**
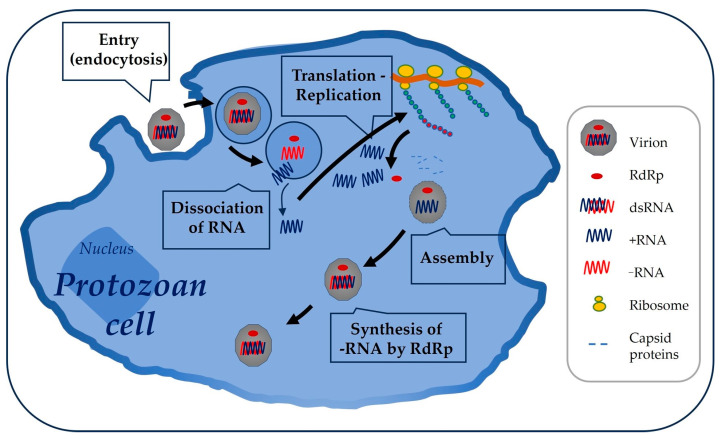
**Replication cycle.** In the *Totiviridae*, viruses replicate in the cytoplasm of the host cell. For this purpose, the virion can enter the protozoan cell by an endocytosis-mediated process. Transcription of the dsRNA genome by the viral polymerase occurs inside the virion so that the dsRNA is never exposed to the cytoplasm. The dsRNA contained in the cytoplasmic vesicles is cleaved into two ssRNAs. The positive-sense RNA (+RNA) leaves the vesicle and is translated by the ribosomes, generating the capsid proteins and RdRp. These begin to assemble, and RdRp polymerizes the complementary negative-sense RNA (−RNA), both in the vesicles and in the newly formed viral particles, a process that is repeated several times, resulting in new mature virions.

The replication cycle of *Totiviridae* appears to follow this general strategy: Positive-sense RNA transcripts are produced from the parental dsRNA conservatively and released into the cytoplasm, where they are either translated into viral proteins or encapsidated to serve as a template for viral replication. A single round of negative-strand synthesis using a full-length positive-strand RNA as a template is thought to generate the duplex RNA genome in newly assembled virions [25,26]. Neither reverse transcriptase activity nor integration activity have been reported for these viruses. A question remains regarding their functionality. However, sequences of the capsid and the RdRp genes have been identified in many of them. It has been previously described that in class I *Totiviridae,* the second ORF originates from a −1 or +1 frameshift jump caused by the heptamer motif, a pseudoknot, and stem-loop structures within the overlap region, which delays translation. The proteins that are synthesized assemble and pack positive-sense RNA along with POL. The enzyme polymerizes the complementary strand of the RNA, thus forming dsRNA again, and the cycle restarts. Consequently, dsRNA is never exposed in the cytoplasm to be detected by the host cell [20,27] (Figure 3). 

As mentioned before, in *Totiviridae* the new mature virions can either leave the host cell horizontally [7,16,28] (by exocytosis or extracellular vesicles (EVs)) or be transmitted vertically during the replication of the protozoan [29]. *Giardiavirus* may be liberated into the extracellular environment without harming the cell (not by cell lysis) and infect other *Giardia* trophozoites [6]. This requires maturation and extrusion. Maturation of the capsid (and possibly also of POL) involves post-translational processing by *Giardia*-specific cysteine proteases that remove 32 amino acids of the N-terminus [30] during post-translational processing, which facilitates viral protein maturation [30]. It is hypothesized that these 32 amino acids may represent the GLV membrane-permeable peptide, and as only 2 are charged, they facilitate entry into the cell [2], closing the replication cycle. 

Like most other members of the *Totiviridae* family, TVV and LRV do not appear to have the molecular machinery necessary to exit or enter their protozoan host. However, extracellular transmission between *T. vaginalis* cells via EVs has recently been proposed as a possible transmission pathway [31,32], as occurs in LRVs [33], and, thus, they resist environmental conditions and are transmitted into other host protozoan cells [7,34]. Indeed, it has been reported that pathogenic protozoa can use secreted EVs to communicate with each other or interact with distal host cells. These are particles naturally released by the cell, bound by a lipid bilayer, and unable to replicate, as defined by the International Society for Extracellular Vesicles (ISEVs). Interestingly, viruses have been found inside EVs [35]. *Leishmania* EVs are used as viral envelopes by the *Leishmaniavirus,* facilitating transmission. Similarly, *T. vaginalis* cells release TVV particles into EVs. Also, several groups have described two distinct types of EVs secreted by *Giardia* [36].

## 3. Flagellated Protozoans of Health Relevance

### 3.1. Giardia-Giardiavirus

Giardiosis is a diarrheal disease caused by *Giardia lamblia*, also known as *G. intestinalis* or *G. duodenalis*. It is a flagellated protozoan parasite that inhabits the gastrointestinal tract of humans and many other mammals. The disease has a worldwide distribution, with a higher incidence in poor countries with a lack of sanitary conditions. Transmission usually occurs by the ingestion of cysts via contaminated water, food, or the fecal–oral route [37]. The life cycle of *Giardia* has two different phases: the intestinal flagellated trophozoite and the infective cyst, which is highly resistant to environmental conditions. After the ingestion of infective cysts in contaminated food or water, the excystation process begins after reaching the stomach. The acidity of the stomach triggers the breakdown of the *Giardia* cyst wall. Excysting trophozoites complete cell division and then trophozoites search for suitable sites for intestinal attachment and colonization using flagellar motility. This parasitic phase has a ventral suction disc through which it adheres to the microvilli of intestinal cells, where they feed, secrete cysteine proteases, and multiply actively, all of which, together, cause damage to the intestinal mucosa, favoring the appearance of clinical symptoms [38]. The descent of the trophozoites into the large intestine determines the exposure to an environment of a higher pH, bile, and lactic acid concentration that favors some of the trophozoites to differentiate into cysts (the encystation process), which are excreted in feces [20]. The cyst wall gives the parasite the necessary protection against environmental factors to survive for weeks–a month in cool, moist environments. This facilitates the dissemination of parasites and the infection of new hosts [39,40]. *Giardia* parasites can be spread by ingesting as few as 10 cysts, making it easily transmissible [41]. Most infected individuals are symptomatic. Even if the disease becomes chronic, the parasites adhere to the human intestinal wall and cause losses in digestion and nutrient absorption. Unlike *G. lamblia* infection in humans, most *Giardia* infections in dogs are asymptomatic [42]. No effective and approved vaccine against giardiosis has been developed. The first-line treatment is metronidazole; however, there are other therapeutic options, such as tinidazole, which requires only one dose and has fewer adverse effects [39].

The *Giardia* genus is divided into eight genetic groups, termed assemblages A–H [43]. Each assemblage is generally associated with a particular host specificity [38,39], of which A and B are the most infectious to humans and can spread zoonotically [44]. The other six assemblages (C–H) are found almost exclusively in non-human hosts, such as beavers, cats, dogs, and cattle [39]. A recent report has also described human infections by assemblage E [45]. The degree to which a mammal is affected by giardiosis is highly variable. The outcome of the severity of the parasitic infection depends on many variables derived not only from the parasite but also from the mammalian host. Indeed, one of the unsolved questions of giardiosis is to determine which factors control whether the disease progresses asymptomatically or symptomatically. Kraft et al. [46] identified several putative *Giardia* virulence factors and other non-parasitic aspects that could be decisive in the progression of giardiosis. These factors include the presence of the *Giardiavirus* GLV (*Giardia lamblia* virus) and its potential contribution to the virulence of its parasitic host [46]. 

GLV was first reported in 1986, when a linear dsRNA molecule was observed in nucleic acid extracts of *G. lamblia* trophozoites obtained by Dr. D. G. Lindmark. Further analysis of this dsRNA showed it to be a genome from a small virus that infects this protozoan. The virus found was named GLV, referring to the host it infects [6]. Currently, according to the International Committee on the Taxonomy of Viruses (ICTV), GLV belongs to the genus *Giardiavirus* of the family *Totiviridae*. GLV is a non-enveloped, icosahedral virus with a diameter of 36 nm, which has a non-segmented dsRNA genome of approximately 6.2 kb with two ORFs on one of the genomic RNA strands, encoding the CP (ORF1) and the viral RdRp (ORF2) [47]. GLV is specific to *Giardia* trophozoites since it does not infect other protozoa [48]. In a recent study, the authors updated the phylogenetic relationships of the family *Totiviridae*. The phylogenetic analysis revealed a clade that includes Giardiaviruses and a large diversity of new Totiviruses, which infect arthropods, protozoa, and mollusks. This expanded *Giardiavirus* clade comprises two monophyletic groups, one of which includes GLV grouped with viruses infecting arthropods and vertebrates (the GLV-like group), and the other includes the previously proposed *Artivirus* group (the IMNV-like group). The results reinforce the considerable structural diversity of hosts and genomes within the *Totiviridae* family, especially when the extended *Giardiavirus* clade is considered. Despite their substantial differences, especially in the comparison of the ORF1 structure, phylogenetic analysis revealed that the GLV-like and IMNV-like groups present close evolutionary relationships, thus sharing the same common ancestor [49]. 

In addition to GLV, *Giardia canis* virus (GCV), has also been described [50]. GCV was isolated in 2001 from different strains of *G. canis*. Although GCV does not yet have a fully defined classification, phylogenetic analysis of RdRp gene sequences demonstrates that it is clustered with GLV. In fact, the striking similarity in genome organization and sequence identity (they share more than 94% identity), as well as the closer phylogenetic proximity of their hosts, may indicate that GLV and GCV share a common evolutionary origin [3]. Studies on the physical characteristics of GCV are still needed but based on its high genomic sequence similarity to GLV, it can be assumed that both have a similar structure. Sequence alignment studies of the cDNAs of GLV, GCV, and *Saccharomyces cerevisiae* virus L-A (ScV-L-A), one of the best-known viruses of the *Totiviridae* family [51], *Leishmania* RNA virus (LRV), and *Trichomonas vaginalis* virus (TVV) indicate that, despite the similar organization of their viral genomes, GLV and GCV share very little overall sequence identity with any of these viruses whose genomic sequences have been fully determined. Therefore, GLV and GCV are not closely related evolutionarily to these other viruses. The capsid of GLV has an outer diameter of approximately 485 Å, making GLV (and probably also GCV), the largest *Totiviridae* capsid analyzed to date [2]. Since *Giardia* species are distributed worldwide affecting hosts in both developing and developed countries, it is expected that GCV and GLV follow their respective hosts with a worldwide distribution [3]. 

Other viruses infecting *Giardia* have been described in the literature but are less studied. A study recently reported a novel unclassified viral sequence (GdRV-2) in at least one isolate of *G. lamblia* assemblage E, co-infected with GLV. GdRV-2 is unrelated to *Giardiavirus* and lacks an independent ORF coding for a putative capsid protein, which likely suggests that it may belong to a new unclassified family of capsid-less viruses (such as the ssRNA(+) Narnaviruses and dsRNA Hypoviruses in fungi and dsRNA Endornaviruses in fungi and plants) [52]. Recently, Kinsella et al. identified three groups of ssDNA viruses (informally referred to as CRESS virus [53]) associated with protozoan parasites [54]. One of these groups of viruses infects *G. lamblia* (specifically, groups A2 and B) and could constitute the *Vilyaviridae* family, with an estimated prevalence of 27% [53]. Recently, experts have referred to this virus as the *Giardia*-associated CRESS DNA virus (taxonomy ID: 2766565) [55].

#### 3.1.1. Endosymbiotic Relationship

*Giardiavirus* can specifically infect *Giardia* sp., but it is not lethal to its host [56]. Several studies have reported that 36–47% of *G. lamblia* isolates from human samples were positive for GLV RNA [57,58]. The degree of resistance of *Giardia* isolates to GLV infection is variable. In contrast with resistant isolates, it appears that only susceptible parasites have a surface GLV-binding receptor (which has not yet been identified in detail, but its ligand is an epitope of the viral capsid) that determines the entry of the virus by endocytosis [48,52,59]. Remarkably, another study confirmed that 70% of *G. lamblia* virus-positive isolates have the virus receptors on their cell surface, biochemical evidence of their phenotype of susceptibility to GLV infection [50]. Like some mammalian cell-borne viruses, GLV appears to exploit the *Giardia* endocytic pathway. Once binding to the plasma membrane, the virus accumulates in the peripheral vacuoles [60], where a low pH environment allows the release of its genetic material. As mentioned above, members of the *Totiviridae* family package the positive strand of RNA into the subviral particle, and then RNA polymerase generates the negative strand with which the bases are base-paired to produce dsRNA, without being exposed to the cytoplasm and remaining invisible to the host cell [20]. It has been described that the microRNA of the virus (GLV miARN1), encoded by the RdRp gene, is involved in GLV replication within the cytoplasm of the trophozoite, reaching a viral load of about 500,000 copies, leading to the end of *Giardia* growth [27,56]. It is noteworthy that GLV is extruded into the culture medium without damaging host cells (a harmless phenotype) and infects other susceptible trophozoites [7,18,48]. Similar to the mature virions of other genera of the *Totiviridae* family, Giardiaviruses are transmitted both extracellularly (exocytosis) and during replication. Recent studies have detected RNA in EVs released by *Giardia* isolates [61]; however, further studies are necessary to confirm the presence of GLV in them. *Giardiavirus* transcripts electroporated into *Giardia* trophozoites can generate a progeny of infective mature, contagious viral particles [18]. Other in vitro studies have also shown the ability of infectious RNA transcripts derived from GCV cDNA to transfect trophozoites of virus-free *Giardia* isolates derived from dogs. The results showed that GCV infected the parasitic cell, multiplied, and packaged into new mature infectious viral particles that were eventually released from the host cell [50]. Other authors have found that the virus can egress the trophozoite by budding at the plasma membrane via exosomes or microvesicles, or even after lysis of the trophozoites [50,52]. It is also unknown whether GLV can remain inside the parasite when it transforms from a trophozoite into a cyst or vice versa. In fact, most studies have not yet described its presence in cysts [2,20], although this possibility could be indicated by a study that seemed to detect *Giardiavirus* in cysts from fecal samples of different animal species [62]. Therefore, in the absence of further conclusive evidence, it appears that when a mammalian host ingests *Giardia* cysts, these are free of GLV, but even contaminated food or water may also carry GLV virions, and it is within the mammalian intestine that the free GLV enters by endocytosis into the parasitic trophozoite phase [2,63].

#### 3.1.2. Endosymbiotic Modulation of Virulence and Immune Response

The interaction of the virus endosymbiont with *Giardia* sp. trophozoites does not seem to be associated with an increase in the virulence of the parasite, unlike the *Totiviridae* viruses LRV and TVV that infect other flagellated protozoa (*Leishmania* sp. and *Trichomonas vaginalis*, respectively), and its effect on the severity of the disease caused in a human host [8,27,58,64]. According to some authors, it is necessary to characterize several GLV strains from naturally infected *G. lamblia* isolates and to perform detailed transcriptomic studies in order to understand whether endosymbiosis determines an alteration in the phenotypic clinical profile of *Giardia* [52]. Recently, a scientific group succeeded in constructing an experimental model of *Giardiavirus*-infected *G. lamblia* (termed the VIG strain) based on the *G. lamblia* WB strain (ATCC30957) and found that the trophozoites of the VIG strain proliferated significantly faster than those of the virus-free *G. lamblia* WB strain. In addition, the cyst formation rate of the VIG strain was significantly lower than that of the *G. lamblia* WB strain [56]. That is, it appears that at a certain viral load, the growth rate of the trophozoites seems to be proportionally reduced [52,58]. A crucial feature in the horizontal transmission of GLV is its heat-resistance capacity making the viral capsid very stable (compared with LRV and TVV virions), as shown in its different capsid structure [2]. This fact explains the near absence of viral dsRNA release that could interact with the TLR of intestinal surface epithelial cells, and, consequently, the proinflammatory response of GLV could be lower, as reported in the literature [7].

**Figure 4:**
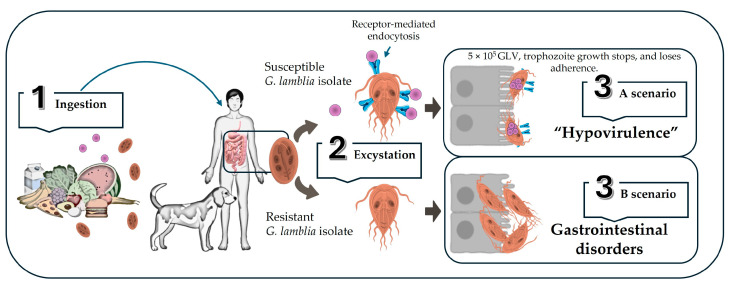
**Schematic model of *G. lamblia* GLV+ interaction in a mammalian host.** The process is initiated (1) by the ingestion of food or water contaminated with GLV-free virions and *G. lamblia* cysts. Once the cysts reach the small intestine, (2) excystation occurs, releasing the parasitic trophozoites. At this point, depending on the susceptibility of the *G. lamblia* isolate, two possible scenarios are shown. In a susceptible isolate (3A), GLV enters the trophozoite by receptor-mediated endocytosis, establishing endosymbiosis that may limit the ability of the trophozoites to adhere to the gastrointestinal epithelium and further growth. This is associated with a hypovirulent clinical phenotype of giardiosis. In a resistant isolate (3B), the trophozoite lacks GLV-binding receptors, there is no endosymbiosis, and the trophozoite adheres to intestinal cells, producing a conventional pathogenesis of giardiosis.

There are also studies suggesting that there is a decrease in virulence (hypovirulence) of *Giardia* trophozoites when they are infected by GLV. Experiments performed with axenic cultures of some strains of *G. lamblia* seem to indicate that upon reaching a viral load of 5 × 10^5^ GLV, trophozoite growth stops, and although the virus does not lyse it, it loses its adherence and ability to replicate [65]. In this situation, it is worth asking whether the effect of the modification of the parasitic phenotype (loss of adherence) leads to an advantage for the mammalian host [8]. This adhesion defect on intestinal cells may reduce the virulence of *Giardia* sp. so that in mammals infected with GLV+ *Giardia* sp., the course of the disease may be more benign (a hypovirulent clinical phenotype of giardiosis) compared with those infected with GLV-free *Giardia* trophozoites (Figure 4).

All these aspects make it a fascinating study model to elucidate the evolutionary relationship between viruses and protozoa [66]. The induction of efficient immune mechanisms in humans or animal models infected with *G. lamblia* seems essential in the development of a mixed Th1/Th2 response, as well as the Th17 response in infection control. The cytokine TNF-α is an important pleiotropic cytokine involved in host defense and inflammation. IL-6 is a multifunctional cytokine involved in the promotion of T-cell differentiation into Th17 cells, as well as in T-cell and B-cell responses. IL-6 can regulate B-cell maturation (thus also participating in Th2 responses) and induce a shift in the antibody type to IgA in response to *Giardia* infection, an antibody that is important at the intestinal mucosal level in eradicating both *G. muris* and *G. lamblia* infection [67,68]. Within this framework of modulation of the immune system of a mammalian host, in vitro studies of murine macrophages stimulated with *Giardia* GLV+ trophozoites showed a stronger inflammatory response (higher levels of IL-6, TNF-α, and IL-12 p40 secretion) compared with *Giardia* GLV- trophozoites, mediated by TLR9 [69]. In addition, in several experimental animal models of giardiosis, the induction of a Th17 immune response or a strong Th1 response has been strongly associated with protection against giardiosis. Thus, the proinflammatory response (high levels of IL-6, TNF-α, and IL-12 p40) is also important in reducing the *Giardia* trophozoite load and fecal cyst counts [67,68]. This could be related to the acquisition of a more efficient immune response to control the disease when a mammalian host is infected with *Giardia* sp. GLV+ compared with GLV-free *Giardia*. 

### 3.2. Trichomonas-Trichomonasvirus

*Trichomonas vaginalis* and *Tritrichomonas foetus* are parasitic protists that colonize the urogenital tract of humans and cattle, respectively. The first one is the most common non-viral curable sexually transmitted infection (STI), with an estimated incidence of more than 156 million cases worldwide [70]. The latter is the major cause of infertility in cattle, associated with high embryonic mortality in bovines [71], although it can also induce gastrointestinal disease in cats or become a commensal protozoan in swine [72]. In humans, *T. vaginalis* can only be acquired sexually, at which time the parasite attaches to vaginal epithelial cells through its surface LPG and other host receptors, which are still unknown [12]. This infection is associated with a wide range of clinical manifestations, from asymptomatic cases in ~80% of men and 50% of women to more severe inflammatory processes [73,74]. The main signs in female patients are mainly non-specific and include pruritus, vulvitis, vaginitis, or dysuria, with yellow–green malodorous leucorrhea or strawberry cervix (also known as *colpitis macularis)* [74,75]. In men, non-gonococcal urethritis and dysuria are common in those who develop symptoms [74]. During pregnancy, *T. vaginalis* is associated with preterm birth, placental abruption, and pregnancy outcome. This parasite increases the predisposition to acquire other sexually transmitted pathogens, such as human immunodeficiency virus (HIV), human papillomavirus (HPV), herpes virus, bacterial vaginosis, *Chlamydia trachomatis*, *Neisseria gonorrhoeae*, and *Treponema pallidum* [74,76]. Trichomonosis has also been associated with the development of cervical and prostate cancer due to the inflammatory response triggered by *T. vaginalis* at the genitourinary level [77,78]. 

#### 3.2.1. Endosymbiotic Relationship

The virulence factors associated with these parasitic STIs are better characterized in the human parasite *T. vaginalis*, although several factors have been identified in both genera. For instance, surface molecules, such as adhesins or lipoglycan, are essential for parasite adherence to host cells. Moreover, cytotoxic effects due to cysteine proteases and other molecules are key in the pathogenesis of both infections [76,79].

The recent discovery pointing out that *Trichomonas* is capable of harboring other microorganisms [80] has been of great relevance in understanding the complexity and versatility of this parasite. Regarding the ability to host viruses (TVV), its transmission among trophozoites seems to be vertical during asexual reproduction by binary fission of the parasite, since TVV does not have the molecular machinery to infect new trichomonads [26]; however, the presence of the viral capsid protein in EVs released by *T. vaginalis* isolates suggests another alternative mechanism for horizontal transmission between trophozoites [31] entering *T. vaginalis* via endocytosis [81]. Once inside, TVV can be detected in the cytoplasm, associated with the Golgi complex or the plasma membrane, with a heterogeneous size (33 to 200 nm) and shape (filamentous, cylindrical, and spherical) [81]. In relation to other trichomonads, virus-like particles (VLPs) have been detected in *Tritrichomonas foetus* [82], but no dsRNA segments or VLP have been identified in *T. gallinae* samples from avian hosts [83]. 

The infection rate of TVV in trichomonads isolates is highly variable, with an estimated overall mean prevalence of nearly 50% [84], ranging from 14% in South Korea [85] to 100% in India [86], as shown in Table 1. To date, five types of TVV have been described (TVV1–TVV5) [87,88,89,90,91]. The most prevalent strains are TVV1 and TVV2 [84,90,92], while there are no data on TVV5's prevalence, as it was recently discovered [91]. Thus, a single *T. vaginalis* cell can be infected by one, two, or even four different TVV isolates [90]; however, the implications of multiple co-infections in the modulation of the parasite's virulence are unknown. 

In an attempt to establish a clear grouping of *T. vaginalis* isolates based on biomolecular features, two-type population distribution has been detected: type 1, which is more frequently found in isolates carrying TVV (*T. vaginalis* TVV+) and linked with greater pathogenicity, and type 2, which is associated with metronidazole resistance [94,113]. These two clades have been detected using different molecular tools, such as RAPD [113], microsatellites, single-nucleotide polymorphisms [94], and multi-locus sequence typing (MLST) [92].

#### 3.2.2. Endosymbiont Modulation of Virulence and Immune Response

Several studies affirm that the endosymbiotic relationship between *Trichomonas* and these *Totiviridae* viruses influences the virulence of this sexually transmitted pathogen. The presence of VLP in different isolates of *T. vaginalis* was first reported by Wang et al. in 1985 [87]. Since then, researchers have associated the presence of these virus-encoded dsRNAs with phenotypic variations [112]. TVV, in most cases, has been described as a virulence factor of *T. vaginalis*, altering total protein expression in the protozoan [16]. The protein P270, present in the cytoplasm of negative isolates (*T. vaginalis* TVV−), changed its localization on the surface of the parasite in *T. vaginalis* TVV+ isolates [115,116]. Similarly, a loss of P270 expression on its surface was associated with a loss of TVV [117]. This confers a relevant value to P270 as a diagnostic marker. It has been reported that the presence of TVV causes a complementary action between P270 and cysteine proteinases. Furthermore, the P270 protein, like cysteine proteinases, favors the evasion of the host immune system, but it is also an immunogenic protein that has been associated with an exacerbated immune response leading to increased inflammation [16]. Moreover, the heterogeneous expression profiles of cysteine proteinases have been associated with a potential advantage in host immune evasion by *T. vaginalis* TVV+ isolates [116]. Cysteine proteinases are well-known virulence factors of different parasites, including *T. vaginalis*, with multidisciplinary functions, such as immunoglobulin and complement protein degradation, invasion and cytoadherence to cells of the vaginal epithelium, and cytotoxicity (the degradation and destruction of proteins by hydrolysis), among others [118]. Recent proteomic studies have shown different protein expression profiles between TVV+ and TVV− isolates. Among the more than 50 proteins involved, the differential expression of certain adhesins, heat shock proteins, enzymes involved in metabolic pathways, and ribosomal proteins stand out [119].

Although TVV cannot replicate in human cells, it can amplify proinflammatory responses in the host [15] when the immune system detects the presence of virions or dsRNA through TLR3 [12]. How TVV virions or viral dsRNA interact with vaginal epithelial cells (first, at the plasma membrane and then, along the endocytic pathway) is still unknown. One possibility is that since the TVV capsid is composed of 120 icosahedral-shaped subunits with large channels, it may facilitate the release of viral dsRNA that could be taken up by human cells. Another related possibility mentioned below is due to metronidazole therapy. Another option is, for example, that extracellular or endosomal proteases digest the TVV capsid and release the viral genome, which is exposed to enter the endocytic uptake pathway and interact within the endosome with TLR3 to determine the observed immunoinflammatory responses that contribute to the pathogenesis of human trichomoniasis and its complications [15].

**Figure 5:**
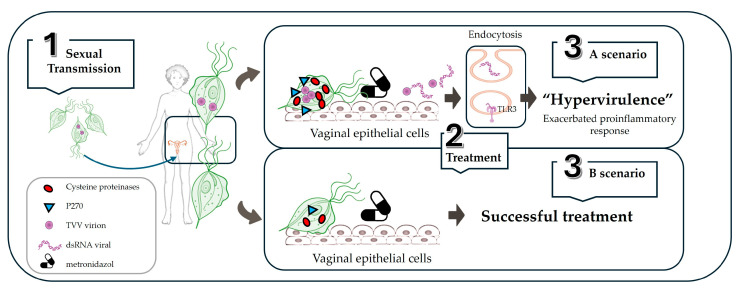
**Schematic model of *T. vaginalis* TVV+ interaction in a human host.** The trophozoites are sexually transmitted (1) and adhere to the genitourinary epithelium cells through the interaction of their surface LPG and adhesins, among other molecules, with different host ligands. The parasite triggers virulence factors that determine the clinical course of the disease (i.e., P270 and cysteine proteinases), with an increased inflammatory reaction in the presence of TVV. After the initiation of antiprotozoal treatment with metronidazole (2), two scenarios are shown depending on the presence or absence of the TVV endosymbiont. If the infection is caused by *T. vaginalis* TVV+ (3A), treatment with metronidazole results in the death of the protozoan and the release of virions and viral genetic material available for endocytic entry into the cells of the vaginal epithelium and interaction with TLR3 of the host endosome. This results in a hypervirulent phenotype of the disease, accompanied by its complications, which may exacerbate the clinical signs and are particularly relevant in pregnant women. However, if the infection is caused by *T. vaginalis* TVV− (3B), treatment with metronidazole is successful, and the disease is controlled.

Based on the above, changes in the expression of certain proteins and the induction of proinflammatory responses due to the presence of TVV could favor greater virulence in those infected trichomonads and, therefore, affect the severity of the clinical illness [32]. The research conducted by Fraga et al. reported a significant association between the presence of TVV and moderate–severe symptoms in comparison with *T. vaginalis* TVV− isolates [98], especially those of *T. vaginalis* TVV2+ [99]. This association is in consonance with other studies where the isolates harboring the virus were obtained from symptomatic patients with specific clinical symptoms, such as vaginal discharge, dysuria, or erythema, and showed a high virulence in the murine intraperitoneal model compared with *T. vaginalis* TVV− [102]. In this sense, the association of the type 1 *T. vaginalis* population with the presence of TVV and pathogenicity is plausible [92]. Nevertheless, other studies have not detected a statistical association between clinics and the presence of this endosymbiont [28,86,107,114], not considering TVV an adequate virulence marker [86]. Regarding resistance to metronidazole, nearly 10% of clinical *T. vaginalis* isolates are estimated to be resistant to the drug of choice [120]. The potential contribution of TVV to an increased susceptibility to metronidazole in *T. vaginalis* isolates has also been proposed, but the relationship between TVV and metronidazole resistance remains controversial. In a study conducted by Malla et al. [86], the research group observed that isolates with TVV were more likely to be susceptible to the reference drug, consistent with the two-type population structure in *T. vaginalis*, where type 2 is linked to higher metronidazole resistance and also to a lower presence of TVV [92,94,113]. Nevertheless, other larger studies report no association [28,96]. Further studies with a high number of isolates are necessary to shed light on the potential impact of this endobiont on the clinical manifestations or regarding drug resistance. What is remarkable in terms of accurate diagnosis and the choice of the most appropriate treatment is that in the case of infection caused by *T. vaginalis* TVV+, antiprotozoal therapy with metronidazole may exacerbate the inflammatory response. The reason is that the lethal action of metronidazole on parasites releases virions and dsRNA that remain accessible to human host TLR3 recognition (Figure 5), amplifying inflammatory damages [10,15,64]. In this scenario, the identification of VLPs after drug treatment in both *T. vaginalis* and *T. foetus* samples [12,82] opens a new debate on whether in certain cases (e.g., during pregnancy) it is convenient or not to treat trichomonosis, as the immune recognition of viruses can increase the risk of clinical complications [12]. 

### 3.3. Leishmania-Leishmaniavirus

Leishmaniases are a group of vector-borne zoonoses caused by protozoan parasites of the genus *Leishmania* and are transmitted amongst mammalian hosts by phlebotomine sandflies (an insect vector of the genus *Phlebotomus* in the Old World and *Lutzomyia* in the New World). These zoonoses have a worldwide distribution, although, like other neglected diseases, they are mainly influenced by environmental changes and socioeconomic factors, such as poor housing and sanitary conditions, malnutrition, or population movements [121]. Leishmaniases show four main clinical presentations: cutaneous (CL), visceral (VL), post-kala-azar dermal (PKDL), and mucocutaneous leishmaniosis (MCL). The clinical presentation depends on the infective *Leishmania* sp. and the immune status of the mammalian host. There are currently no vaccines available, and treatment options rely on drugs with toxic side effects, which require parenteral administration and are susceptible to resistance, reducing their efficacy. Furthermore, control strategies for these diseases represent an action track in line with the One Health Program Priority Area [122,123]. 

VL presents with fever, weight loss, and hepatosplenomegaly and may have neurological manifestations. If untreated, it has a fatality rate of over 95%. PKDL can cause erythematous or hypopigmented macules, papules, nodules, and patches. CL patients present with plaques, nodules, and/or ulcers and, in the case of MCL, symptoms manifest on the mucous membranes of the nasal and oral cavities and surrounding tissues. These forms of leishmaniosis leave visible disfiguring lesions and lifelong scars on the skin. Cutaneous lesions have been linked to social stigma that could potentially lead to isolation and self-stigma. Thus, in some cultures, women are considered unfit for marriage or are separated from their children when they have this disease. There is a considerable health impact because patients with leishmaniosis may have a higher risk of mental illness, psychosocial morbidity, and reduced quality of life [124]. It has even been reported that dogs naturally infected with canine LV, as well as murine models of LV, show neuroinflammation [125,126]. Also, people co-infected with *Leishmania*–HIV are more likely to develop disseminated forms of leishmaniosis, as well as high relapse and mortality rates. In fact, as of three years ago, high prevalences of *Leishmania*–HIV co-infection have been reported in 45 countries. The highest rates of *Leishmania*–HIV co-infection were found in Brazil, Ethiopia, and India [127].

Parasites of the genus *Leishmania* have a digenetic life cycle, alternating between a motile extracellular phase, flagellated promastigotes, and intracellular amastigotes that multiply inside the phagocytic cells of the mammalian host [128]. 

Evasion of macrophage immune sentinel is a well-established strategy in intracellular parasites. *Leishmania* sp. has developed various survival strategies to evade or modulate the immune defense to reside in the hostile environment of host macrophages. In a recent review, the authors discuss in detail the variety of pathogenicity scenarios of *Leishmania* infection. TLRs are expressed on the membranes of endosomes and lysosomes or on the surfaces of macrophages and dendritic cells. After PAMP recognition, TLRs induce a cascade of signaling pathways that promote the synthesis of antileishmanial products in macrophages and dendritic cells, such as proinflammatory Th1 cytokines. However, *Leishmania* parasites can survive within the infected macrophages by manipulating the activation mechanisms of their TLRs. Thus, TLRs play a functional duality and specific role in response to the ligands expressed by the pathogen, intervening either in the elimination of *Leishmania* or in the magnification of the pathology [129]. A recently described virulence factor is the release of *Leishmania* EVs in the digestive tract of sandflies, which ensures a more efficient infection of the mammalian host and exacerbates skin pathologies [130].

#### 3.3.1. Endosymbiotic Relationship 

The first report of infection of *Leishmania* strains by a virus occurred in 1974, when VLPs were observed in the cytoplasm of *Leishmania hertigi* [131], isolated from the prehensile-tailed porcupine, and currently classified as *Paraleishmania hertigi* [132,133]. The term VLP has been used to describe a number of structures with uncharacterized viral morphology found in biological samples [134]. Moreover, it should be noted that attempts to purify viral particles and their nucleic acids from *P. hertigi* cultures have failed. A similar case of an unconfirmed viral nature occurred in 1980, when VLPs were observed in *Endotrypanum* sp., a trypanosomatid parasite that infects sloth erythrocytes and can be transmitted by sandflies in South and Central America [135]. In those years, reports about VLPs were mostly based on transmission electron microscopy (TEM), and their viral nature could not be confirmed. Advances in molecular biology and, more specifically, in diagnostic techniques over the years have finally made it possible to obtain complementary results to confirm this type of finding [133]. Thus, the existence of endoprotozoal *Leishmaniavirus* of the *Totiviridae* family was discovered almost three decades ago [136,137,138], but the knowledge of their role in disease progression is still incomplete. The described LRVs have been classified into two types: LRV1 (present in the New World *Leishmania* species of the subgenus *Viannia*) and LRV2 (present in the Old World *Leishmania* species of the subgenus *Leishmania*). According to phylogenetic studies, the genetic distance between New and Old World parasite species is similar to the distance between LRV1 and LRV2 virus species [139]. Thus, the complete nucleotide sequence of the RNA-dependent RNA polymerase (RdRp) gene shows high diversity between LRV1 and LRV2 (with an overall sequence identity level below 40%) [140]. From an evolutionary perspective, LRV is assumed to have been present before the divergence of both *Leishmania* subgenera and thus has followed different evolutions due to the geographic separation of *Leishmania* parasites. A decade ago, the first observation of LVR2 in Africa, together with the earlier description in Turkmenistan in Asia, reinforced the theory of independent coevolution, as LRV2-positive strains had not yet been detected in the New World. Since then, until now, LRV2 has been detected in *L. aethiopica* [141], *L. major* [140,142,143], *L. infantum* [144], and *L. tropica* [143]. Interestingly, a recent finding is the first report of LRV2 in the New World (Brazil), having detected LRV2 in *L. infantum* [145]. The authors recommend further full study before interpreting the consequences of this result (the size of the amplified fragment was too small, at 300 bp) in view of a possible reconsideration of the hypothesis of independent evolution. 

Although much remains to be elucidated, several studies have focused on determining the benefits and costs of endosymbiosis for both LRV and *Leishmania*, including the ultimate impact on the infection of a mammalian host. Among the potential advantages acquired by *Leishmania* species causing mucocutaneous leishmaniosis (belonging to the subgenus *Viannia*, *L. braziliensis*, and *L. guyanensis*) due to their endosymbiont association with LRV are the following: (i) the parasite increases its level of resistance against the hostile environment that the mammalian macrophage generates through oxidative stress, (ii) the parasite increases its transmission since it survives during chronic infection to disseminate in the mammal as a parasitic metastasis, and (iii) the parasite enhances its resistance to antileishmanial drugs [29]. The question regarding the advantages for the virus is obvious: whether viral persistence depends on maintaining a copy number compatible with host survival. Interestingly, the generation of *Leishmania* hybrids in both laboratory cultures and experimental hybrids generated in phlebotomine sandflies demonstrates the ability of *Leishmania* to hybridize. This suggests that these mechanisms of genetic exchange may be driven by a sexual process. In relation to these questions of genetic exchange, one might consider whether this event is frequent under natural conditions and whether the horizontal transmission of LRV is feasible [146]. Moreover, results from other studies support that parasite gene flow and hybridization have increased the frequency of parasite–virus symbioses, a process that may change the epidemiology of leishmaniosis in different countries [147]. In addition to reports of horizontal transmission [148,149], vertical transmission appears to be the predominant mode of viral transmission resulting in the overall coevolution of *Leishmania* and LRV [133]. Sustained viral persistence over time could result in the excessive production of viral proteins, leading to host death and, ultimately, selective pressure to eliminate the virus. Thus, it is reasonable that LRV employs regulatory mechanisms to maintain persistent infection. Among them, LRV may inhibit its replication by overexpressing the capsid protein. Another way in which the virus can interact with its protozoan host is through the excision of host RNA. Sequence analysis of the *Leishmania* rRNA and endoribonuclease cleavage sites of New World and Old World viruses has identified several regions of the rRNA sequence that possess similarities to the cleavage site. According to this hypothesis, when the virus copy number increases, transcripts are cleaved in addition to *Leishmania* rRNA, and a slower more regulated form of translation occurs, resulting in a persistent infection compatible with parasite survival [25].

#### 3.3.2. Endosymbiont Modulation of Virulence and Immune Response

Parasitic virulence factors target important host functions and mechanisms [150]. Among them, it is interesting to consider parasitic ligands that modulate TLR receptor-mediated signaling, especially TLR2, TLR4, and TLR9 [129]. The effects of such activation are complex and depend on the nature of the TLR, the cell type, the *Leishmania* species, and the timing in which those events occur [151]. TLR2-mediated recognition of the LPG on the surface of *L. major*, *L. mexicana*, and *L. aethiopica* triggers the development of a protective immune response by increasing ROS and NO production, while in *L. amazonensis* and *L. braziliensis*, recognition of the LPG (of a different thickness than the other species) activates TLR2 to promote the persistence of infection [152]. Other studies demonstrated the importance of TLR4-mediated iNOS activation in *L. major* clearance, and TLR4 has also been shown to detect LPG in visceral species and stimulate inflammatory signaling, leading to parasite clearance [153]. Moreover, TLR9 is the only TLR member that is solely associated with host resistance against *Leishmania* infection by inducing IL-12 secretion, which ultimately leads to IFN-γ production by natural killer cells. In fact, there are CpG-based therapeutic approaches to activate TLR9 and produce IL-12 [154]. TLR3 recognizes viral dsRNA and promotes IFN-α/β expression, which enhances antiviral protein synthesis (AVP) activity [155,156]. An essential finding that is the focus of our present review is that activation of mammalian host TLR3 upon interaction with LRV1 endosymbiotic virus dsRNA during infection by *Viannia* subgenus species (*L. guyanensis* and *L. braziliensis*) induces an uncontrolled hyperinflammatory response that increases parasite load and disease pathogenesis [9,129,157]. Comparative studies in murine experimental models have described how infection with *L. guyanensis* parasites carrying LRV1 (LgyLRV1+), unlike LgyLRV1−, leads to low NO production that allows parasite dissemination and metastasis (developing severe lesions), mediated by elevated IL-17 synthesis in lymph nodes draining the initial site of infection [158]. In addition, considering that TLRs were first discovered in insects, direct studies on the influence of this virus on the relationship between *Leishmania* and sandflies would be of great interest. In fact, some authors suggest the hypothesis that the viruses invade and persist as mere parasitic elements rather than providing any advantage to their trypanosomatid hosts [159].

**Figure 6:**
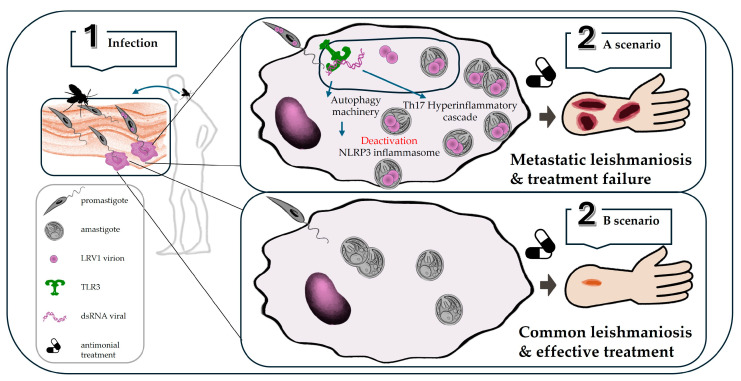
**Schematic model of *L. guyanensis* LRV1+ interaction in a mammal host.** Infectious promastigotes are released by the phlebotomine sandfly bite (1) during a blood meal and enter target phagocytic cells (macrophages). Depending on whether the promastigotes carry LRV1 or not, two possible scenarios are shown. In the case of *L. guyanensis* LRV1+ infection (2A), promastigotes transform into amastigotes inside the cell phagosome, and viral RNA can be released, which interacts with TLR3 inside the phagosome and triggers both the hyperinflammatory Th17 cascade (leading to metastatic lesions) and the activation of the autophagic machinery, resulting in the deactivation of the inflammasome platform, thus favoring parasitic persistence associated with treatment failure. In cases of *L. guyanensis* LRV1- infection (2B), the disease course progresses in a conventional manner, and disease control can be achieved with successful antimonial treatment.

Several studies have reviewed various immunological mechanisms related to the development of the most aggressive cutaneous and mucocutaneous leishmaniosis caused by *Leishmania* species of the subgenus *Viannia* LRV1+ in the Americas [34,151,160]. Moreover, some authors clarify the role in the vertebrate host of *Leishmania* EVs previously released in the digestive tract of sandflies that may carry LRV1, further enhancing the metastatic and hyperinflammatory phenotype [130]. Taking all the above into account, the following model graphically shows the crucial steps in the LRV-mediated signaling pathway during *Leishmania* infection (Figure 6).

Once the insect bite occurs, LRV1 can access the vertebrate either within *Leishmania* or within EVs to be phagocytosed by macrophages and engulfed in a phagosome. Within the phagosome, TLR3 activation occurs either by the interaction of free LVR1 dsRNA or LRV1 contained in the parasite’s extracellular vesicles. This triggers TLR3/TRIF signaling, leading to the induction of inflammatory cytokines (TNF-α and IL-12) to promote inflammation, although type I IFNs such as IFN-β are also induced, which activates the autophagy machinery [161] in infected macrophages, interfering with the parasite multiplication-limiting the NLRP3 inflammasome molecular platform [162,163]. As a result, pathological aggravation and parasitic persistence and dissemination occur, respectively. Thus, LRV1 correlates with the induction of metastasis in patients, promoting mucocutaneous leishmaniosis [34]. However, there are studies that argue that there is no evidence for the involvement of LRV2 in determining the outcome of leishmaniosis [164]. 

Whether the presence of LRV1 in these *Leishmania* species of the Americas can determine a possible therapeutic failure, contradictory studies have been reported [165,166]; however, most of them support the hypothesis that there is a higher risk of therapeutic failure and disease relapse in patients infected with *L. guyanensis* and *L. braziliensis* LRV1+, even accompanied by the appearance of metastatic lesions [34,167,168,169]. Likewise, LRV2 has been isolated from a patient infected with *L. infantum*, who did not respond to Glucantime^®^ treatment, which could be suggestive of a possible role of LRV2 in drug resistance [164]. In addition, another recent study suggested a possible relationship between the severity of clinical presentations observed in *L. aethiopica* infections and the presence of an LRV in some of these parasitic strains. Furthermore, they showed that a virus related to *L. major* LRV2 was widespread and could evoke cytokine responses like those previously observed with *L.* (*Viannia*) LRV1. This sets the stage for future studies on the role of LRV2 in the severity and nature of human leishmaniosis [141]. Considering the high prevalence of up to 70% of *Leishmania* LRV+ isolates in certain endemic regions and the association of the virus with treatment failure, disease control efforts should also focus on VLRs, as they represent a crucial target [170].

### 3.4. Trypanosoma and Other Suspected VLPs

In addition to *Leishmania*, *Trypanosoma* is another genus of notable medical importance as human pathogens, both within the family Trypanosomatidae. The genus *Trypanosoma* includes *T. cruzi* and *T. brucei* as species causing human parasitosis: Chagas disease (also known as American trypanosomosis) and sleeping sickness (also known as human African trypanosomosis), respectively. Although cases of *T. rangeli* infection have also been described in the American population [171], it is a non-pathogenic protozoan and does not cause human illness [172]. It should be noted that, unlike the cases of endosymbiosis between double-stranded RNA viruses of the family *Totiviridae* and the pathogenic flagellated protozoan species mentioned in previous sections, there is only one description in *T. cruzi* and some evidence of suspected endosymbiosis with species of the genus *Trypanosoma* [8]. However, recent research [173] has investigated the presence of VLPs in the different stages of *T. cruzi* by electron microscopy, resulting in the first description of both cytoplasmic enveloped and non-enveloped VLPs in *T. cruzi* epimastigotes. In a study, the researchers analyzed the presence of VLPs in different stages of the parasite by electron microscopy. Thus, two different classes of viral particles were detected in the cytoplasm of *T. cruzi* epimastigotes. On the one hand, enveloped VLPs with a diameter of 48 nm were found, and the authors argued for the possibility that they were acquired under laboratory conditions during the feeding of *T. cruzi*-infected *Triatoma* specimens with pigeon blood harboring RNA viruses belonging to the *Flaviviridae* family [173]. On the other hand, non-enveloped VLPs (with a diameter of 32 nm) structurally compatible with those of the *Totiviridae* dsRNA family were also detected. The study exposed the complexity of understanding how *T. cruzi* parasites acquired these VLPs under natural conditions, either from the arthropod vectors or from the mammalian hosts. Similar to what has been described for other vector-borne protozoa, such as *Plasmodium*, in the case of *T. cruzi*, its vectors (i.e., triatomines) could harbor non-enveloped RNA viruses transferred to the parasite as it passes through the arthropod gut during its life cycle development [8]. However, the biological implication of this finding remains to be established, requiring studies focused on the evaluation of whether the presence of a putative *Totiviridae* in *T. cruzi* epimastigotes modifies the virulence of the parasite.

## 4. Concluding Remarks and Future Perspectives

Viruses are probably the most abundant “biological entities” on Earth and obligatory parasites. Unable to synthesize their own proteins, they use the cellular machinery to translate their messenger RNA into proteins. Hyper-parasitism is the rule in nature, and viruses are present in all cells including protozoa. Unlike common mammalian viruses, the genomic structural diversity in *Totiviridae* generates a variable viral replication cycle, although with common stages, such as the absence of nuclear genomic integration, self-replication, assembly, and the regulation of its protozoan host to achieve its main objective: viral persistence without damaging the protozoan cell. Besides the possible role played by endosymbiotic viruses as drivers of evolutionary changes via the horizontal transfer of genetic material among protozoa (not addressed in our review) or the unraveling of speciation processes [159], they are of relevance in the balance of parasitic protozoa–mammalian host relationships. Since the discovery of RNA viruses in *Acanthamoeba* over 50 years ago, PPVs have been described in different groups including Apicomplexa (*Plasmodium* and *Leucocytozoon*) and flagellates [64,174]. Thus, a triangular matryoshka-type infection relationship (between the virus, parasitic protozoa, and mammalian host) emerges [7,8], which is of paramount importance in the epidemiology, pathogenicity, and control of human protozoal infections. The reported cases vary from those of apparent hypovirulence, observed in *G. lamblia* sp. GLV+ hypervirulence, in which the activation of the TLR3 of the mammalian cell by the TVV or LVR-1 genome determines immunopathological alterations with lesions, parasite survival, and possible therapeutic failures [7,10,168]. Our knowledge of PPVs is still fragmentary, and more research is needed to unravel the intricacies of this three-sided relationship. Studies could provide a framework to understand the interaction between the PPV, parasitic protozoa, and the mammalian host [175] and could provide useful tools to limit the extension and severity of human infections. The development of efficient control methods against flagellated protozoa of medical relevance depends on the availability of early and efficient diagnostic methods; knowledge of their virulence, in which endosymbiotic viruses play a relevant role; and the modulation of the immune response of the mammalian host. Biotechnological advances and previous results regarding other frequent human viruses suggest that the potential of PPVs can be exploited to know the epidemiology of protozoal infections [147] and to develop ad hoc immunological and therapeutic strategies. There are some lines of research with potential applicability based on PPVs: (i) The induction of direct parasiticidal activity using endosymbiont viruses seems to be a good possibility, which is now only applicable to *Giardia* sp. (at least for some isolates of the parasite GLV+), so that overloading the parasite with GLV causes cell growth arrest and probably cell death. This potential strategy requires the evaluation of potential adverse effects and the adequacy of the infectious dose [7,64]. (ii) Genetic editing of the endosymbiont virus to achieve molecular manipulation of the parasite is another possibility. The classical CRISPR-Cas gene-editing system has already been used to successfully make mutations in *Giardia*, *Plasmodium*, *Trichomonas*, and *Leishmania*, but it does not appear to be able to easily cross cell membranes [176]. An alternative is to disrupt parasite survival by vectorizing the delivery of nucleic acid sequences. One way to overcome this obstacle would be to use VLPs for in vivo delivery of the nucleic acid cargo. (iii) Another possibility is VLP-based chemotherapy. VLPs have an empty viral protein envelope but do not contain the viral genome and are non-infectious and safe. This Trojan Horse approach, if properly designed and formulated, represents a promising platform for the development of treatments against protozoa for therapeutic or immunological applications [175,177]. The outer surface can also be genetically or chemically optimized [64]. In the case of TTV and LRV, VLPs can be stripped of their nucleic acid content to avoid an adverse human host response to viral dsRNA and efficiently loaded with the appropriate drug (metronidazole or antimonial). (iv) Promising results using antiviral immunization have been obtained in mice with the LRV1 capsid, reducing parasite pathogenicity and generating protective immunity [170,178]. Manipulation of the immune response of an infected mammalian host requires careful, perhaps individualized, consideration of protozoan infection and endosymbiont PPVs. Despite the suggestive results, further research in predictive surrogate models with well-characterized protozoal strains is needed to develop new tools for the effective control of protozoal infections [7,8,64].

## Figures and Tables

**Table 1:** Identification of dsRNA virus (TVV) in *T. vaginalis* isolates.

Country	Type of Sample	TVV Presence	TVV Type Isolates	Co-Infections % (n) *	Ref.
Austria, Brazil, China, Czech Republic, Estonia, Slovakia, Sweden, and USA	NR	44.4% (8/18)	NR	NR	[93]
Australia, Chile, India, Italy, Mexico, Papua New Guinea, Southern Africa, and USA	Vaginal swabs	30.3% (67/221)	NR	NR	[94]
Czech Rep., Slovakia, USA, China, Brazil, Estonia, Sweden, and Austria	Vaginal samples + 3 ATCC isolates	40% (8/20)	NR	NR	[95]
Brazil	Urine samples + 2 ATCC isolates	90.9% (30/33)	TVV1 = 24 TVV2 = 9 TVV3 = 11 TVV4 = 3	36.7% (11/30)	[96]
Cuba	Vaginal samples	55% (22/40)	NR	NR	[97]
Cuba	Vaginal exudates	55% (22/40)	NR	NR	[98]
Cuba	NR	56.7% (21/37)	TVV1 = 19 TVV2 = 15	14.3% (3/21)	[99]
Cuba	Vaginal samples	100% (3/3)	TVV1 = 1 TVV2 = 2	NR	[100]
Egypt	Vaginal swabs	35% (7/20)	NR	NR	[101]
Egypt	Vaginal swabs	20% (8/40)	TVV2 = 5 TVV4 = 3	0% (0/8)	[102]
India	Vaginal swabs and urine samples	100% (30/30)	NR	NR	[86]
Italy	NR	50% (24/48)	TVV1 = 17 TVV2 = 19 TVV3 = 13 TVV4 = 2	75% (18/24)	[103]
Iran	Vaginal discharge and urine samples	17.4% (8/46)	TVV1 = 8	NR	[104]
Iran	Vaginal swabs	50% (4/8)	TVV1 = 4	0% (0/4)	[105]
Iran	Vaginal samples	44.4% (4/9)	TVV1 = 4 TVV2 = 1 TVV3 = 1	25% (1/4)	[106]
Kenya	Vaginal swabs	43.5% (10/22)	TVV1 = 9 TVV2 = 6 TVV3 = 4 TVV4 = 3	90% (9/10)	[107]
Korea	NR	14% (4/22)	NR	NR	[85]
Netherlands	Cervicovaginal + urine samples	50.4% (60/119)	TVV1 = 42 TVV2 = 26 TVV3 = 34	51.7% (31/60)	[92]
Philippines	Vaginal swabs	18.7% (18/96)	TVV1 = 12 TVV2 = 12 TVV3 = 5 TVV4 = 6	33.3% (6/18)	[108]
Philippines	Vaginal swabs	30.9% (13/42)	NR	NR	[109]
Turkey	Vaginal swabs	16.7% (5/30)	NR	NR	[110]
South Africa	Clinical samples	81.9% (59/72)	NR	NR	[111]
USA	NR	50% (14/28)	NR	NR	[112]
USA	Vaginal swabs and clinical samples	50% (55/109)	NR	NR	[113]
USA	Vaginal swabs and urine samples	75% (21/28)	NR	NR	[114]
USA	Vaginal swabs	40% (142/355)	NR	NR	[28]
USA	Vaginal swabs	100% (5/5)	TVV1 = 5 TVV2 = 3 TVV3 = 3 TVV4 = 4	100% (5/5)	[90]
USA	Vaginal swabs	81.2% (13/16)	NR	84.6% (11/13)	[12]

NR: not reported. * Co-infections: isolates with more than one TVV type.

## Data Availability

The original contributions presented in this study are included in this article. Further inquiries can be directed to the corresponding author.

## Abbreviations

LPG	Lipophosphoglycan
iNOS	Inducible nitric oxide synthase
NO	Nitric oxide
ROS	Reactive oxygen species
TLR	Toll-like receptor
